# Human Brain and Blood N-Glycome Profiling in Alzheimer’s Disease and Alzheimer’s Disease-Related Dementias

**DOI:** 10.3389/fnagi.2021.765259

**Published:** 2021-10-27

**Authors:** Lei Yu, Zhiguang Huo, Jingyun Yang, Helena Palma-Gudiel, Patricia A. Boyle, Julie A. Schneider, David A. Bennett, Jinying Zhao

**Affiliations:** ^1^Rush Alzheimer’s Disease Center, Rush University Medical Center, Chicago, IL, United States; ^2^Department of Neurological Sciences, Rush University Medical Center, Chicago, IL, United States; ^3^Department of Biostatistics, University of Florida, Gainesville, FL, United States; ^4^Department of Epidemiology, University of Florida, Gainesville, FL, United States; ^5^Department of Psychiatry and Behavioral Sciences, Rush University Medical Center, Chicago, IL, United States; ^6^Department of Pathology, Rush University Medical Center, Chicago, IL, United States

**Keywords:** glycosylation, N-glycans, AD/ADRD, cognition, neuropathologies

## Abstract

Glycosylation, the process of adding glycans (i.e., sugars) to proteins, is the most abundant post-translational modification. N-glycosylation is the most common form of glycosylation, and the N-glycan moieties play key roles in regulating protein functions and many other biological processes. Thus, identification and quantification of N-glycome (complete repertoire of all N-glycans in a sample) may provide new sources of biomarkers and shed light on health and disease. To date, little is known about the role of altered N-glycome in Alzheimer’s Disease and Alzheimer’s Disease-related Dementias (AD/ADRD). The current study included 45 older adults who had no cognitive impairment (NCI) at baseline, followed and examined annually, and underwent brain autopsy after death. During about 12-year follow-up, 15 developed mild cognitive impairment (MCI), 15 developed AD, and 15 remained NCI. Relative abundances of N-glycans in serum at 2 time points (baseline and proximate to death, ∼12.3 years apart) and postmortem brain tissue (dorsolateral prefrontal cortex) were quantified using MALDI-TOF-MS. Regression models were used to test the associations of N-glycans with AD/ADRD phenotypes. We detected 71 serum and 141 brain N-glycans, of which 46 were in common. Most serum N-glycans had mean fold changes less than one between baseline and proximate to death. The cross-tissue N-glycan correlations were weak. Baseline serum N-glycans were more strongly associated with AD/ADRD compared to change in serum N-glycans over time and brain N-glycans. The N-glycan associations were observed in both AD and non-AD neuropathologies. To our knowledge, this is the first comprehensive glycomic analysis in both blood and brain in relation to AD pathology. Our results suggest that altered N-glycans may serve as mechanistic biomarkers for early diagnosis and progression of AD/ADRD.

## Introduction

Alzheimer’s Disease and Alzheimer’s Disease Related Dementia (AD/ADRD) affects over 5 million older Americans in 2021, and the number is projected to double by 2050. Clinically, AD/ADRD is characterized by cognitive decline and impairment in multiple cognitive domains. The neuropathologies of AD/ADRD are complex and include Alzheimer’s disease (AD), non-AD neurodegenerative and cerebrovascular conditions that commonly coexist in aging brain (Corrada et al., 2012; White et al., 2016; Boyle et al., 2021). It is increasingly recognized that these pathologies appear years or even decades before the onset of clinical symptoms (Davis et al., 1999; Bennett et al., 2006b; Sonnen et al., 2011). Identifying mechanistic biomarkers is key to early diagnosis and effective prevention or intervention of AD/ADRD. Post-translational modifications (PTMs) represent an attractive mechanism, as many proteins implicated in AD (e.g., β-amyloid and tau) and non-AD neuropathologies (e.g., α-synuclein and TDP43) undergo PTMs (Marcelli et al., 2018; Pajarillo et al., 2019; Schaffert and Carter, 2020).

Protein glycosylation, an enzymatic process that attaches glycans (i.e., sugars) to proteins, is one of the most common forms of PTMs. There are two major types of glycosylation. N-glycans are attached to an asparagine residue of protein, and O-glycans to a serine or threonine residue (An et al., 2009). Glycosylation defects specific to amyloid precursor protein (APP), tau and β-site amyloid precursor protein cleaving enzyme-1 (BACE1) have been implicated in AD. For example, prior studies have shown that aberrant N-glycosylation delays lysosomal degradation of BACE1 and contributes to β-secretase cleavage of APP (Kizuka et al., 2015). Separately, it has been reported that glycosylation occurs before hyperphosphorylation of tau in AD, and N-glycans help to maintain the paired helical filament (PHF) structure of phosphorylated tau (Wang et al., 1996; Liu et al., 2002). A more recent study further reveals that protein N-glycosylation aberrations and alterations of glycoproteomic network are implicated in AD (Zhang et al., 2020). As the population of glycans that occurs at a given protein site is heterogeneous, understanding the role of glycosylation in AD/ADRD requires a detailed knowledge about the structurally distinct glycans and their functions. To date, only a small number of studies have probed glycan profile in relation to AD (Gizaw et al., 2016; Palmigiano et al., 2016). To our knowledge, no study has quantified the change in blood glycan profile over time along with paired brain tissue samples, or has examined the extent to which altered blood and brain glycans are associated with non-AD neuropathologies.

In this study, we extended prior work on N-glycan profiling by conducting a pilot study examining human serum at two time points and post-mortem brain tissue from the same individuals. By leveraging rich clinicopathologic data and prospectively archived biospecimen from two cohort studies of aging and dementia, 45 community-living older adults were selected. All individuals had no cognitive impairment (NCI) at baseline. At death, a third remained NCI, a third developed mild cognitive impairment (MCI) and a third Alzheimer’s dementia. N-glycans in serum samples at baseline, proximate to death, and frozen tissue from dorsolateral prefrontal cortex (DLPFC) were quantified by matrix assisted laser desorption ionization-time of flight mass spectrometry (MALDI-TOF-MS). We described N-glycan characteristics within serum over time as well as cross-tissue correlations between serum and brain, and investigated the N-glycan associations with a suite of cognitive, clinical and pathologic traits of AD/ADRD.

## Materials and Methods

### Study Participants

Biospecimen were obtained from participants in either the Religious Orders Study or Rush Memory and Aging Project (ROSMAP) (Bennett et al., 2018), two ongoing cohort studies of aging, dementia, and other chronic conditions. ROSMAP follows almost identical study design and data collection protocol. In brief, participants were community dwelling older adults free of known dementia at enrollment, who underwent annual uniform structured clinical evaluations that include an interview on medical history, medication inspection, a comprehensive cognitive assessment followed by diagnosis of cognitive impairment and dementia, as well as a blood sample collection. Participation in ROSMAP requires brain donation after death. Both studies were approved by an Institutional Review Board of Rush University Medical Center, and the studies were conducted in accordance with Declaration of Helsinki for experiments involving humans. An informed consent, Anatomical Gift Act for organ donation, and a repository consent were acquired from each participant in this study.

Sera and cortical N-glycan profiles were quantified using biospecimen from 45 eligible ROSMAP autopsied cases. All were NCI at baseline. At death, 15 remained NCI, 15 were diagnosed with MCI and 15 with Alzheimer’s dementia. To investigate change of sera glycan profile over time, we required the time intervals between baseline and proximate to death to be at least 10 years apart. All participants had completed detailed neuropathologic assessments for common neurodegenerative and cerebrovascular conditions.

### Clinical and Cognitive Assessments

Cognition was assessed using a battery of 19 neuropsychological tests which covers 5 domains of episodic memory (7 tests), semantic memory (3 tests), working memory (3 tests), perceptual speed (4 tests) and visuospatial ability (2 tests) (Wilson et al., 2002). Individual test scores were standardized using baseline mean and standard deviation (SD) of the entire cohorts and then averaged across the tests to yield a summary score for global cognition. Annual global cognitive scores were used for capturing change in cognition over time. Details on decision rules guiding the diagnosis of Alzheimer’s dementia were described previously (Bennett et al., 2006a). Briefly, cognitive testing results were rated by a computer algorithm, and the ratings were reviewed by a neuropsychologist to determine the presence of cognitive impairment. Diagnosis of dementia and its likely etiology was provided by a clinician after reviewing all clinical records. Alzheimer’s dementia diagnosis follows the NINCDS/ADRDA criteria (McKhann et al., 1984) which require a history of cognitive decline and impairment in memory and at least one other domain. Older adults who were cognitively impaired but did not meet the clinical criteria for dementia were classified as MCI.

### Neuropathologic Evaluations

At autopsy, the brain was removed, weighed and cut into 1 cm slabs, following a standard procedure (Schneider et al., 2003). Slabs from one hemisphere were rapidly frozen at −80°C and slabs from the other hemisphere were fixed for at least 3 days in 4% paraformaldehyde. A systematic uniform neuropathologic evaluations was conducted on the fixed tissue, blinded to all clinical data (Boyle et al., 2021). Quantitative and semiquantitative measures of common age-related neurodegenerative and cerebrovascular conditions, including AD, Lewy bodies, limbic predominant age-related TDP-43 encephalopathy (LATE), hippocampal sclerosis, macroscopic infarcts, microinfarcts, cerebral amyloid angiopathy, atherosclerosis and arteriolosclerosis, were recorded. Multiple AD pathologic measures were obtained (Supplementary Methods). Briefly, a continuous measure for global burden of AD pathology summarizes counts of neuritic plaques, diffuse plaques and neurofibrillary tangles across 5 regions (Bennett et al., 2003). Pathologic AD diagnosis was determined according to the NIA-Reagan criteria using moderate or high likelihood of AD threshold. Using molecular specific antibodies, we quantified average β-amyloid load and separately PHFtau tangle density across 8 regions (Kapasi et al., 2021). Presence of Lewy bodies was identified in 7 regions using α-synuclein immunostaining (Schneider et al., 2007). LATE were quantified in 7 regions using immunohistochemistry with a phosphorylated monoclonal TAR5P-1D3 anti-TDP43 antibody and summarized into 4 stages (Nelson et al., 2019). Considering the small N in this work, we dichotomized the measure into TDP43 absent or localized in amygdala only vs. more advanced stages where TDP43 inclusion extends to hippocampus, entorhinal or neocortex. Hippocampal sclerosis refers to severe neuronal loss or gliosis in CA1/subiculum of the hippocampus, and was determined using hematoxylin and eosin (H&E) staining (Nag et al., 2015).

Macroscopic infarcts were identified at gross examination using fixed slabs and digital pictures of both hemispheres, and confirmed histologically (Schneider et al., 2003). Microinfarcts in at least 9 regions was identified using H&E staining (Arvanitakis et al., 2011). Meningeal and parenchymal vessels in 4 regions were examined for β-amyloid deposition. Average scores across the regions were summarized into a semi-quantitative rating, i.e., none, mild, moderate or severe. Vessels in Circle of Willis were visually inspected for atherosclerotic plaques (Boyle et al., 2019). Small arterioles of the anterior basal ganglia were assessed for concentric hyaline thickening of vessel walls. Severity of atherosclerosis and arteriolosclerosis were rated on a semi-quantitative scale, i.e., none, mild, moderate or severe (Arvanitakis et al., 2016). Similar to LATE, we dichotomized these ordinal measures into none/mild vs. moderate/severe in subsequent statistical analysis.

### N-Glycome Quantification by Matrix Assisted Laser Desorption Ionization-Time of Flight Mass Spectrometry

Blood N-glycans: Blood was drawn at annual evaluation and aliquots of serum and plasma were stored in a −80°C freezer. Serum samples at baseline and proximate to death were used for N-glycan quantification. All MALDI-TOF-MS analyses were conducted by the Glycomics Core of Beth Israel Deaconess Medical Center at Harvard Medical School (Supplementary Methods). Briefly, serum samples were lyophilized, and N-glycans were released. Permethylated N-glycans were purified and extracted. MS data was acquired on a Bruker UltraFlex II MALDI-TOF Mass Spectrometer. Post-data acquisition analysis was conducted using mMass (Strohalm et al., 2010). MS signals that have a signal/noise ratio of at least 4 and match an N-glycan composition were included. The resulting sera glycan intensities were normalized using a global scaling (Vallejos et al., 2017). Individual sera glycan intensity was divided by the mean of sera glycan intensities within each sample, and then multiplied by the grand mean of glycan intensities across all the samples. The normalized intensities were log2 transformed to reduce skewness and facilitate interpretation.

Brain N-glycans: 20–100 mg frozen tissues from DLPFC were dialyzed after suspended in lysis buffer. The material was lyophilized, and treated with dithiothreitol and iodoacetamide. After lyophilization, samples were resuspended in TPCK-treated trypsin solution and processed for purification. Released N-glycans were collected, pooled and lyophilized. The permethylation, MS data acquisition and post-acquisition analysis follow a procedure similar for sera N-glycans (Supplementary Methods).

### Statistical Analysis

Demographic, clinical and neuropathologic characteristics were described using analysis of variance (ANOVA), Chi-squared test, Fisher’s exact test, or non-parametric Kruskal-Wallis test, as appropriate. Pairwise correlations, visualized using histograms and heatmaps, illustrate interrelationships between N-glycan profile within and across tissues. N-glycan clusters, for sera and cortical tissue separately, were identified using an oblique principle component analysis with iterative splitting (Harris and Kaiser, 1964). Briefly, starting with a single cluster, the algorithm splits the cluster if the second eigenvalue reaches a pre-specified threshold of λ = 0.7, and the splitting stops when the second eigenvalue of each cluster is no greater than the threshold. Assignment and reassignment of individual N-glycan to a corresponding cluster was determined by the ratio of within-cluster correlation (squared) relative to between-cluster correlation (squared). Component scores for each cluster were computed as the weighted average of N-glycans within each cluster.

To assess the extent to which N-glycans act as potential markers for AD/ADRD, we interrogated individual sera N-glycans at baseline when all individuals were cognitively unimpaired. For each sera N-glycan quantified, linear mixed effects models examined its baseline level in relation to subsequent change in cognition; Cox proportional hazards models examined its association with the risks of developing MCI and separately Alzheimer’s dementia, and linear or logistic regression models examined the N-glycan association with neuropathologies at autopsy with individual pathological indices as separate outcomes. Next, by leveraging N-glycans quantified at both baseline and proximate to death, we estimated change in sera N-glycans over time as the level difference between the two time points. We then replaced baseline sera N-glycan measures with the level differences in the analyses to examine change in sera N-glycans in relation to AD/ADRD traits. Finally, we examined the associations of cortical N-glycans with AD/ADRD neuropathologies using a series of regression analyses.

All the analyses were done using SAS Software version 9.4 and R program version 3.6.3. All statistical models were controlled for the demographics including age, sex and education. Statistical significance was determined using false discovery rate (FDR) level of *q* < 0.05. Considering the small sample size and the exploratory nature of this work, we also reported signals of nominal significance (raw *p* < 0.05) with consistent directions of association across multiple AD/ADRD traits.

## Results

### Participants’ Characteristics

On average, participants were 76.1 (SD: 6.5) years of age at baseline and had 17.9 (SD: 4.2) years of education. Approximately 70% (*N* = 32) were females, and all were Whites. The mean age at death was 89.5 (SD: 4.2) years. The mean time interval between the two blood draws was 12.3 years (SD: 2.1) and the mean time interval between the last blood draw and death was under a year (SD: 0.7).

By design, all the cases were NCI at baseline and at death a third remained NCI, a third had developed MCI and a third Alzheimer’s dementia, over an average of just over 12 years of follow-up for all three groups. Differences in demographic, clinical and neuropathologic characteristics between the 3 groups are reported in Table 1. Briefly, individuals who developed MCI or Alzheimer’s dementia tended to be older and had poorer cognition. We did not find group difference in sex, education, time interval between the 2 blood collections or time interval between the last blood collection and death. As expected, there were significant group differences in neuropathologic burdens. In particular, individuals who developed MCI or Alzheimer’s dementia had more severe PHFtau tangles, Lewy bodies, hippocampal sclerosis, LATE and macroscopic infarcts. Further, cases with cognitive impairment or dementia also had more mixed pathologies.

**TABLE 1:** Demographic, clinical and neuropathologic characteristics.

	NCI (*N* = 15)	MCI (*N* = 15)	Alzheimer’s dementia (*N* = 15)	*p*
Age (baseline), years	72.2 (6.6)	76.7 (5.8)	79.3 (5.2)	0.008^§^
Age (death), years	85.6 (6.6)	89.8 (5.8)	93.0 (5.6)	0.006^§^
Female	10 (66.7%)	11 (73.3%)	11 (73.3%)	0.898*
Education, years	18.3 (4.5)	17.5 (3.0)	17.9 (5.1)	0.898^§^
MMSE (baseline)	30 (29–30)	29 (27–29)	29 (28–29)	0.009^†^
MMSE (proximate to death)	29 (28–30)	26 (25–30)	13 (6–21)	<0.001^†^
Time between 2 blood draws, years	12.4 (2.4)	12.0 (2.0)	12.8 (2.1)	0.556^§^
Time between last blood to death, years	1.0 (0.5)	1.1 (0.5)	0.7 (1.0)	0.452^§^
Number of neuropathologic conditions	1 (1–3)	2 (1–3)	4 (4–5)	0.005^†^
Alzheimer’s disease	8 (53.3%)	9 (60.0%)	12 (80.0%)	0.283*
β-amyloid load	3.46 (0.51–6.36)	2.07 (0.52–3.41)	5.67 (1.30–8.49)	0.186^†^
PHFtau Tangle density	1.87 (0.81–4.46)	2.82 (1.54–6.27)	4.75 (2.36–13.9)	0.022^†^
Lewy bodies	2 (13.3%)	6 (40.0%)	9 (60.0%)	0.030*
Hippocampal sclerosis	1 (6.67%)	0 (0%)	6 (40.0%)	0.002^Δ^
LATE	2 (13.3%)	7 (46.7%)	10 (66.7%)	0.012*
Chronic macroscopic infarcts	1 (6.7%)	1 (6.7%)	10 (66.7%)	< 0.001^Δ^
Chronic microinfarcts	3 (20.0%)	4 (26.7%)	5 (33.3%)	0.912^Δ^
Cerebral amyloid angiopathy	4 (26.7%)	5 (33.3%)	4 (26.7%)	1.000^Δ^
Atherosclerosis	6 (40.0%)	5 (33.3%)	4 (26.7%)	0.741*
Arteriolosclerosis	3 (20.0%)	4 (26.7%)	4 (26.7%)	1.000^Δ^

*Statistics reported are mean (standard deviation), median (interquartile range), or N (%).*

*Alzheimer’s disease: moderate or high likelihood of AD diagnosis per NIA Reagan criteria. LATE, Limbic-predominant age-related TDP-43 encephalopathy. NCI, no cognitive impairment, MCI, mild cognitive impairment.*

^§^Analysis of variance; ^†^Kruskal-Wallis test; *Chi-squared test, ^Δ^Fisher’s exact test.

### Baseline Sera N-Glycans

**FIGURE 1:**
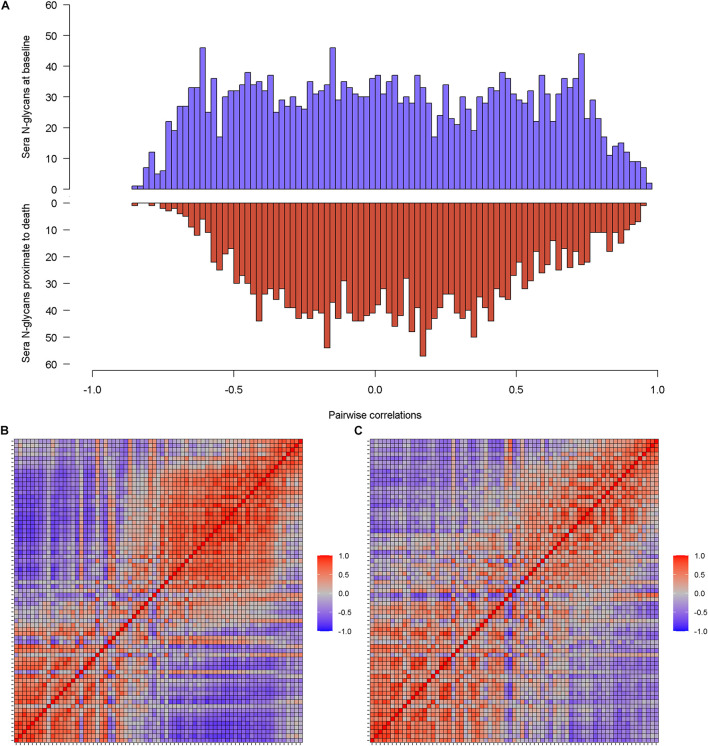
Illustrates the correlations of individual sera N-glycans at baseline and separately proximate to death. **(A)** Is the histograms of pairwise correlations for sera N-glycans at baseline (blue) and proximate to death (red). **(B,C)** Are the heatmaps that further visualize the correlation patterns of sera N-glycan at baseline and proximate to death.

A total of 71 N-glycans were detected and quantified in baseline serum (Supplementary Table 1), of which over 80% were either sialylated or fucosylated N-glycans (Supplementary Figure 1A). Pairwise correlations between baseline sera N-glycans show a relatively uniform distribution (Figure 1A blue). 60% of all pairs were correlated at FDR *q* < 0.05. Within individual N-glycans group, high-mannose N-glycans were highly correlated with each other (Pearson’s r from 0.61 to 0.90). The proportion of correlated pairs between 49 sialylated N-glycans (54.6%) was lower than all pairs of baseline sera N-glycans. Similar results were observed for 33 fucosylated N-glycans (57.8% correlated pairs) and separately 6 IgG non-fucosylated N-glycans (53.3% correlated pairs). These results suggest that, for baseline sera N-glycans, within-group correlations were no stronger than between-group correlations. Separately, the heatmap reveals blocks of N-glycans that are highly correlated at baseline (Figure 1B). An oblique principle component analysis partitioned these 71 baseline sera N-glycans into 13 clusters, which explained 79.4% of the variability (Supplementary Figure 2).

### Sera N-Glycans Proximate to Death

The same set of sera N-glycans (*N* = 71) was quantified in serum samples proximate to death. The distribution of pairwise correlations between N-glycans proximate to death shows a relatively higher peak around the null, compared to baseline (Figure 1A red). Consequently, only 44.2% of the sera N-glycan pairs were correlated proximate to death. Similar to the baseline sera N-glycans, all the high-mannose N-glycans were positively correlated (Pearson’s r between 0.64 and 0.93). Correlated pairs for sialylated N-glycans, fucosylated N-glycans, and IgG non-fucosylated N-glycans were 40.2, 51.2, and 66.7%, respectively. Notably, with the exception of sialylated N-glycans, the proportion of correlated pairs within group was higher than overall correlated pairs. The blocks of correlated sera N-glycans proximate to death (Figure 1C) are sparse compared to baseline. A larger number of sera N-glycan clusters (*N* = 16) were detected, which explained 81% of the variability (Supplementary Figure 2). We observed little resemblance between the clusters at baseline and those proximate to death, suggesting that the clustering structure of baseline sera N-glycans is likely not preserved proximate to death.

The two sets of sera N-glycans measures came from blood collections that were on average 12 years apart. We examined the correlations of the same sera N-glycans between baseline and proximate to death. A majority of the sera N-glycans show a weak correlation between the two time points (Supplementary Figure 3). The median Pearson’s r was 0.24 (interquartile range 0.10–0.34). Sera N-glycans that show relatively high correlation (Pearson’s *r* > 0.5) between baseline and proximate to death include (Hex)4(HexNAc)5(Fuc)1(NeuAc)1, (Hex)4(HexNAc)5(NeuAc)1, (Hex)5(HexNAc)5(Fuc)1(NeuAc)1 and (Hex)7(HexNAc)6(NeuAc)1.

### Change in Sera N-Glycans

**FIGURE 2:**
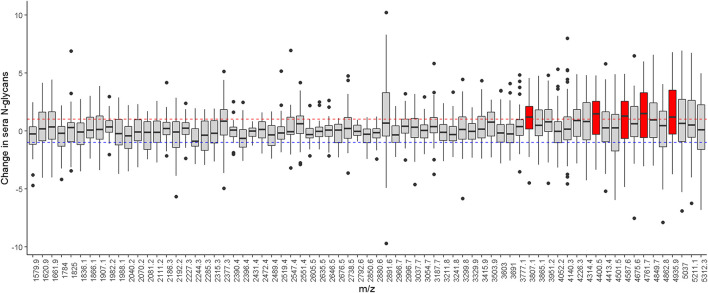
Illustrates the level difference of sera N-glycans between baseline and proximate to death. Individual N-glycans are shown as m/z ratios on the *x*-axis. Horizontal dotted lines represent ± onefold change. Sera N-glycans that passed these thresholds are highlighted in red.

With repeated measurement of sera N-glycans from the same individuals, we examined change in sera N-glycan profile by taking the difference of individual N-glycan levels between baseline and proximate to death. A majority of the sera N-glycans had mean fold changes less than 1 between baseline and proximate to death (Figure 2). The mean fold change of individual sera N-glycans was centered at 0.19 (SD: 0.50, Range: −0.62 to 1.52). Five sera N-glycans with similar compositions had mean fold changes greater than 1, i.e., (Hex)7(HexNAc)5(NeuAc)3, (Hex)7(HexNAc)6(Fuc)2(NeuAc)3, (Hex)7(HexNAc)6(Fuc)1(NeuAc)4, (Hex)7(HexNAc)6(Fuc)2 (NeuAc)4 and (Hex)7(HexNAc)6(Fuc)3(NeuAc)4, all of which were elevated proximate to death compared to baseline.

### Cortical N-Glycans

A total of 141 N-glycans were quantified using brain tissue from DLPFC (Supplementary Table 2). Similar to sera N-glycans, almost all cortical N-glycans were sialylated or fucosylated (Supplementary Figure 1B). The pattern of pairwise correlations between cortical N-glycans was different from that of sera N-glycans, such that the distribution was skewed to the left, suggesting that a majority of the pairs were positively correlated (Supplementary Figure 4A). Two thirds (66.6%) of the cortical N-glycan pairs were correlated at FDR *q* < 0.05. The proportions of correlated pairs for high-mannose, sialylated, and fucosylated cortical N-glycans were 90.0, 68.4, and 82.6%, respectively, suggesting that overall more pairs of cortical N-glycans were correlated within group than that of sera N-glycans. The heatmap also highlights a more pervasive positive correlation pattern between cortical N-glycans (Supplementary Figure 4B). A total of 16 clusters were identified using an oblique principal component analysis, which explained 82.9% of the variability (Supplementary Figure 4C).

Forty six N-glycans were present in both sera and cortical brain tissue. Overall, we did not observe strong cross-tissue correlations of these N-glycans (i.e., sera proximate to death vs. brain cortex). The Pearson’s r ranged from −0.39 to 0.32, with half showing positive correlation coefficients. Notably, N-glycans with lower m/z ratios tended to have inverse cross-tissue correlation than those with higher m/z ratios (Supplementary Figure 5).

### Associations of Baseline Sera N-Glycans With Alzheimer’s Disease and Alzheimer’s Disease-Related Dementias

**FIGURE 3:**
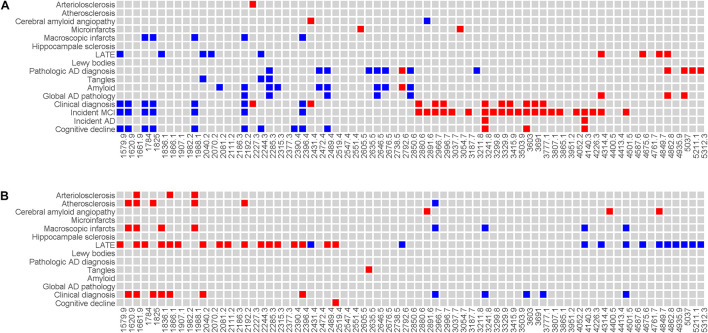
Illustrates the associations of baseline sera N-glycans **(A)** and level difference of sera N-glycan **(B)** with AD/ADRD. Individual N-glycans are shown as m/z ratios on the *x*-axis. Clinical and neuropathologic AD/ADRD outcomes are shown on the *y*-axis. Associations with nominal statistical significance are highlighted (in red if the direction of association is positive and in blue if the direction of association is negative).

We conducted a series of analyses to examine the associations of baseline sera N-glycans with cognitive, clinical and neuropathologic traits of AD/ADRD (Figure 3A). Overall, the results revealed an interesting pattern such that baseline sera N-glycans with lower m/z ratios were associated with slower cognitive decline, lower risk of cognitive impairment or Alzheimer’s dementia, and less neuropathologies. The expression levels of top baseline sera N-glycans associated with clinical diagnosis were compared by cognitive status (Supplementary Figure 6). Further, multiple baseline sera N-glycans show consistent associations with cognitive and clinical AD/ADRD outcomes. Briefly, higher baseline levels of 7 sera N-glycans, including (Hex)5(HexNAc)2, (Hex)4(HexNAc)3, (Hex)6(HexNAc)2, (Hex)5(HexNAc)3, (Hex)7(HexNAc)2, (Hex)8(HexNAc)2 and (Hex)9(HexNAc)2, were nominally associated with slower cognitive decline as well as lower risk of cognitive impairment or Alzheimer’s dementia. Four of these N-glycans, i.e., (Hex)6(HexNAc)2, (Hex)5(HexNAc)3, (Hex)7(HexNAc)2, and (Hex)9(HexNAc)2, were also associated with lower risk of macroscopic infarcts, and (Hex)5(HexNAc)2 was associated with lower risk of LATE. Higher baseline level of another N-glycan, (Hex)8(HexNAc)2, was inversely associated with both β-amyloid load [B: −0.367, 95% confidence interval (CI): −0.673, −0.061] and macroscopic infarcts [odds ratio (OR): 0.193, 95% CI: 0.052, 0.715]. In addition, 7 sera N-glycans showed nominal associations with multiple AD pathologic indices (Figure 3A). Most of these associations were with β-amyloid, with an exception for (Hex)4(HexNAc)5(Fuc)1, where a higher level was associated with less β-amyloid load (B: −0.579, 95% CI: −0.951, −0.207) and lower PHFtau tangle density (B: −0.378, 95% CI: −0.672, −0.083). Separately, higher levels of 3 N-glycans, i.e., (Hex)6(HexNAc)5(NeuAc)2, (Hex)6(HexNAc)5(NeuAc)3, and (Hex)8(HexNAc)7(NeuAc)2, were nominally associated with faster decline as well as greater risk of cognitive impairment or Alzheimer’s dementia. We did not find an association of these N-glycans with neuropathologies.

Analysis of 13 baseline sera N-glycans clusters highlights 3 clusters including Cluster 7, 11 and 13. Four of the 5 N-glycan members in Cluster 7, i.e., (Hex)4(HexNAc)3, (Hex)5(HexNAc)2, (Hex)6(HexNAc)2, and (Hex)5(HexNAc)3, showed consistent associations with multiple AD/ADRD outcomes at the individual glycan level, as described above. Higher component scores for Cluster 7 were associated with slower cognitive decline, lower risk of developing cognitive impairment or Alzheimer’s dementia as well as less neuropathologies (macroscopic infarcts and LATE). Similarly, higher scores for Cluster 11 (6 N-glycan members) and separately Cluster 13 (5 N-glycan members) were nominally associated with slower cognitive decline and less neuropathologies (Supplementary Table 3). Overall, these observations were consistent with the results from individual glycan analysis, suggesting that select N-glycan members of these clusters protect against neuropathologies.

### Associations of Changes in Sera N-Glycans With Alzheimer’s Disease and Alzheimer’s Disease-Related Dementias

Next, we examined the level difference in sera N-glycans between baseline and proximate to death. Overall, the associations of change in sera N-glycans with AD/ADRD traits were weaker than those observed at baseline (Figure 3B). Distinct from the results for baseline where sera N-glycans with lower m/z ratios were associated with slower cognitive decline, lower risk of Alzheimer’s dementia, and less neuropathologies, elevated levels of these N-glycans between baseline and proximate to death tended to be associated with greater risk of cognitive impairment or Alzheimer’s dementia, and more neuropathologies. The change in 12 sera N-glycans was nominally associated with cognitive status proximate to death. Also, the change in sera N-glycans was associated more with cerebrovascular conditions relative to neurodegeneration. In addition, we observed an intriguing pattern of association for LATE, where change in a large number of sera N-glycans (over 40%) was associated with LATE.

### Associations of Cortical N-Glycans With Alzheimer’s Disease and Alzheimer’s Disease-Related Dementias

**FIGURE 4:**
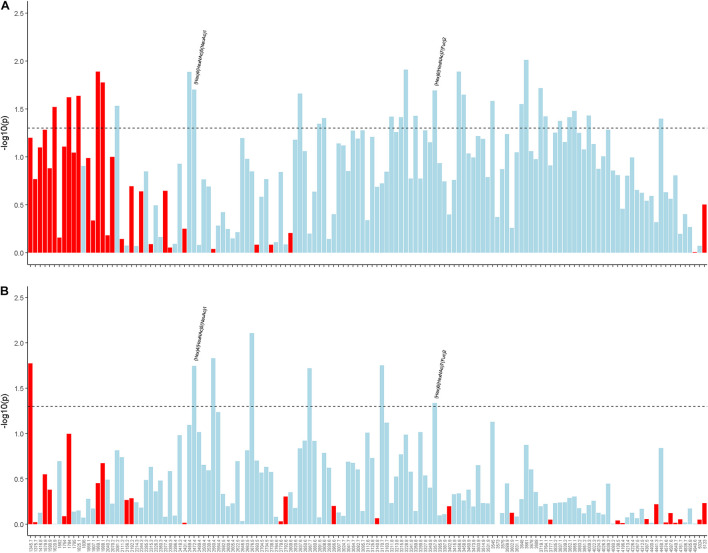
Illustrates the associations of cortical N-glycans with β-amyloid load **(A)** and PHFtau tangles density **(B)**. Individual N-glycans are shown as m/z ratios on the *x*-axis. Statistical significance, represented by –log10 of the *p*-value, is shown on the *y*-axis. The horizontal dotted line represent the nominal significance level of α = 0.05. Red indicates a positive direction of association and light blue indicates a negative direction of association.

Finally, we examined cortical N-glycans in relation to AD/ADRD traits. Two patterns emerged. First, different from baseline sera N-glycans but similar to change in sera N-glycans, those with lower m/z ratios tended to be associated with a greater burden of neuropathologies (Supplementary Figure 7). Second, the associations with AD/ADRD were weaker comparing to the results for baseline sera N-glycans. None of the cortical N-glycan associations with cognitive decline or clinical diagnosis reached nominal statistical significance. However, we observed associations of multiple N-glycans with AD pathologies. Of these, higher levels of two cortical N-glycans, namely (Hex)4(HexNAc)5(NeuAc)1, and (Hex)6(HexNAc)7(Fuc)2, were nominally associated with less β-amyloid load as well as lower PHFtau tangles density (Figure 4). Principal component analysis revealed 16 cortical N-glycan clusters. Of these, higher scores for Cluster 10, including 2 N-glycan members of (Hex)3(HexNAc)4(Fuc)1 and (Hex)3(HexNAc)5(Fuc)1, were nominally associated with lower β-amyloid load (Supplementary Table 4). Of note, higher baseline sera level of (Hex)3(HexNAc)5(Fuc)1 was also associated with lower amyloid pathology.

### Power Calculations

One of the purposes of this pilot is to inform on statistical power for a larger study vs. futility. Therefore, we conducted power calculations to assess minimal sample size required relative to all available sample size in the ROSMAP cohorts, i.e., nearly 1,800 older adults who had died and undergone brain autopsy. For each trait, we estimated sample size required for detecting an N-glycan association with 80% power at a significance level of 0.05, assuming different effect sizes.

For cognitive decline and continuous neuropathologic measures, we estimated the sample size by considering an increase in *R*^2^ based on ordinary linear regression. The sample size to detect an increase in *R*^2^ between 1 and 5% ranged from 150 to 800. For the outcome of incident Alzheimer’s dementia, we considered an increase in hazard ratio (HR) based on Cox regression. Approximately 40% of all ROSMAP autopsied participants developed Alzheimer’s dementia. The sample size to detect an increase between 10 and 40% in HR ranged from 170 to 2,000. Finally, for binary neuropathologic measures, we considered an increase in odds ratio (OR) based on logistic regression. The prevalence of various neuropathologies ranged from approximately 10% (i.e., hippocampal sclerosis) to 60% (i.e., AD). The sample size to detect a 25% increase in odds ratio was between 500 and 2,100. Since our goal of the future project is to identify N-glycans altered in AD/ADRD using all available samples in the ROSMAP cohorts, these power calculations suggest that we will be able to detect important associations.

## Discussion

AD/ADRD poses a major public health threat as our population ages rapidly. Common neurodegenerative and cerebrovascular conditions are the key drivers of AD/ADRD, many of which are histopathologically characterized by deposition of misfolded proteins. Identifying potential causal and regulatory mechanisms involved in protein malformations is pivotal to aging and dementia research. Glycosylation is among the most important and ubiquitous forms of PTMs. The current study employed an unbiased MS-based approach to quantify a large number of N-glycans in peripheral blood at two time points and paired cortical brain tissue from the same individuals who were well characterized. We characterized N-glycan profiles both within and across tissue, as well as longitudinal change in sera N-glycans over time (about 12 years apart). We observed consistent associations of multiple blood or brain N-glycans with cognitive, clinical and neuropathologic traits of AD/ADRD. Our findings expand a very small and emerging literature on the potential utility of peripheral glycans as biomarkers for AD/ADRD, as well as the roles of brain N-glycans in AD/ADRD pathologies.

We are among the first to systematically profile N-glycans in both peripheral blood and brain cortex from the same individuals who were initially free of cognitive impairment but many of whom had developed either MCI or Alzheimer’s dementia. Using a glycoblotting method, a prior study reported 52 sera and 40 cortical N-glycans from a smaller sample (6 pairs of normal and AD subjects) (Gizaw et al., 2016). Here, our MS analysis quantified 71 sera and 141 cortical N-glycans that fall between 1,000 and 6,000 m/z, of which 46 N-glycans were in common. These N-glycans identified were predominantly sialylated or fucosylated. We examined the correlation patterns between these structurally distinct N-glycans within and across tissues. Our data suggest that, compared to serum, cortical N-glycans show a tighter correlation structure and are more closely correlated with each other. Individual pairs of the 46 N-glycans that are common in both tissues on average show relatively low cross-tissue correlation, with a majority of the correlation coefficients within ± 0.3. Interestingly, the expression levels of N-glycans with smaller m/z ratios tend to be inversely correlated between blood and brain comparing to N-glycans with larger m/z ratios.

We quantified sera N-glycans at two time points from the same individuals and examined their longitudinal change over time. Compared to baseline, the correlations among individual sera N-glycans proximate to death were weaker and sparser. The clustering of baseline sera N-glycans appears to be not preserved such that there is little resemblance between clusters at baseline and proximate to death. In addition, we found that over 90% of the 71 sera N-glycans had a mean fold change less than 1, and only a small number of N-glycans (*N* = 5) showed elevation of expression level proximate to death. These observations suggest that, although sera N-glycan levels within a same individual are relatively stable, their correlation structure may vary over time.

Prior studies on glycosylation have primarily focused on key AD-related molecules including APP, α-, β-, and γ-secretases, and tau (Regan et al., 2019). Briefly, previous studies reported that elevated levels of bisecting N-acetylglucosamine (GlcNAc) on BACE1 and knockout of a biosynthetic enzyme for bisecting GlcNAc in mice reduced cleavage of APP by BACE1 and led to a decrease in Aβ plaques (Kizuka et al., 2015). Abnormal N-glycosylation was also implicated in phosphorylation of tau, another pathologic hallmark of AD. One study separated normal tau from hyperphosphorylated and PHF tau in AD brain, and showed that it is glycosylated compared with tau in normal brain (Liu et al., 2002). A few case-control studies investigated N-glycan profiling in relation to AD. One study identified 90 N-glycans from CSF and reported that individuals with AD and MCI show a decrease in sialylation and an increase in N-glycans that bear bisecting GlcNAc (Palmigiano et al., 2016). A separate study reported no significant difference in the major protein N-glycans between AD and normal brain, but the total serum N-glycans expression levels, particularly those of bisect-type, were elevated in AD (Gizaw et al., 2016). This latter result is consistent with our finding that sera N-glycans, rather than cortical N-glycans, overall had stronger associations with AD/ADRD. Here we highlight one N-glycan, i.e., (Hex)3(HexNAc)5(Fuc)1, where higher levels in both baseline serum and brain cortex were associated with lower amyloid burden in brain.

The current work expands findings on the role of structurally distinct N-glycans in AD/ADRD in several important ways. First, all individuals in the study were free of cognitive impairment at baseline, and by design many developed MCI or Alzheimer’s dementia proximate to death. By interrogating baseline sera N-glycans with subsequent AD/ADRD traits, our study informs on the potential utility of N-glycans as biomarkers for early diagnosis of AD/ADRD or disease progression. Notably, baseline sera N-glycans showed the strongest and most consistent associations with AD/ADRD. Multiple N-glycan signals emerged, many of which showed associations with neuropathologic indices at autopsy. For instance, we observed that older adults with a higher (Hex)8(HexNAc)2 sera level at baseline tend to have slower cognitive decline and lower risk for cognitive impairment or Alzheimer’s dementia. A higher baseline level of sera (Hex)8(HexNAc)2 was also associated with lower β-amyloid burden and lower risk for macroscopic infarcts. If confirmed, these results will not only have the potential to nominate peripheral N-glycan biomarkers for AD/ADRD, but could also shed light on the neuropathologic pathways through which altered glycosylation affects AD/ADRD. Second, our results demonstrated that, in addition to AD, aberrant N-glycosylation may also be implicated in non-AD neurodegenerative and cerebrovascular conditions, both of which are common in aging brain. In particular, we showed that multiple sera N-glycans are associated with LATE and macroscopic infarcts. Third, we for the first time assessed the extent to which longitudinal change in sera N-glycans is implicated in AD/ADRD, and showed initial evidence that change in the level of sera N-glycans over time is associated with LATE and vascular conditions. Together, our results suggest that altered N-glycosylation may affect a broad spectrum of AD/ADRD neuropathologies.

Our study has several strengths. By utilizing a high-resolution MALDI-TOF-MS technique and samples from well-characterized older adults who were followed annually until death and underwent brain autopsy, we were able to study a large body of N-glycans in blood samples at different time points along with paired postmortem brain tissue. All individuals were deeply phenotyped for a suite of clinical, cognitive and neuropathologic traits for AD/ADRD. The use of complementary outcome measures and the consistent results increase confidence in our findings. Limitations are also noted. Sample size is small and cases were selected, which may affect the generalizability of our findings. Notably, there is no appreciable difference in major clinical characteristics between subjects included in the current analysis and those in the entire cohorts. Future large-scale studies are warranted to confirm our findings. Separately, the current study did not consider the extent to which other major age-related diseases such as cancer or diabetes are implicated in change of serum N-glycosylation over time.

## Data Availability Statement

N-glycome data used in this study are available upon requests via the corresponding author. ROSMAP data are available via Rush Alzheimer’s Disease Center Resource Sharing Hub at https://www.radc.rush.edu.

## Ethics Statement

The studies involving human participants were reviewed and approved by an Institutional Review Board of Rush University Medical Center, and the studies were conducted in accordance with Declaration of Helsinki for experiments involving humans. An informed consent, Anatomical Gift Act for organ donation, and a repository consent were acquired from each participant in this study. The patients/participants provided their written informed consent to participate in this study.

## Author Contributions

JZ conceptualized the study, collected the N-glycome data, and oversaw the data analysis. LY contributed to study design, conducted data analysis, and drafted the manuscript. ZH conducted data analyses. All authors critically revised the manuscript and contributed to the interpretation of findings for intellectual contents.

## Conflict of Interest

The authors declare that the research was conducted in the absence of any commercial or financial relationships that could be construed as a potential conflict of interest.

## Publisher’s Note

All claims expressed in this article are solely those of the authors and do not necessarily represent those of their affiliated organizations, or those of the publisher, the editors and the reviewers. Any product that may be evaluated in this article, or claim that may be made by its manufacturer, is not guaranteed or endorsed by the publisher.
