# Supplementary material for: Human Brain and Blood N-Glycome Profiling in Alzheimer’s Disease and Alzheimer’s Disease-Related Dementias
Source: Front Aging Neurosci. 2021 Oct 27;13:765259. doi: 10.3389/fnagi.2021.765259 (PMC8579010; doi:10.3389/fnagi.2021.765259)
Supplement: Supplementary file 1 [file Data_Sheet_1.docx]

**Human brain and blood N-glycome profiling in AD/ADRD**

**eMethods**

**Quantification of AD pathologies**

Multiple AD pathologic indices including neuritic plaque, diffuse plaques, neurofibrillary tangles, β-amyloid load and PHFtau tangles density were assessed at autopsy. Counts of neuritic plaque, diffuse plaques, and neurofibrillary tangles were quantified in 5 regions (hippocampus and entorhinal cortex, midfrontal, midtemporal, and inferior parietal cortex) using modified Bielshowsky silver stain. For each AD pathologic index, counts were taken from a 1mm^2^ graticule in the area with greatest density. Region-specific counts were scaled within region and then averaged across regions to obtain a summary score for neuritic plaque, and separately diffuse plaques and neurofibrillary tangles. The three summary scores were then averaged to obtain a continuous measure for global burden of AD pathology.

β-amyloid load and PHFtau tangle density were assessed in 8 brain regions (superior frontal, midfrontal, hippopcampus and entorhinalcortex, inferior temporal, inferior parietal, anterior cingulate, and occipitalcortex) using immunohistochemistry. β-amyloid was detected using a monoclonal antibody. Between 20 and 90 video images of stained sections were captured for a random sample for quantitative analysis of amyloid deposition. Percentage area positive for β-amyloid for each region was square root transformed and averaged across brain regions to obtain a summary measure for β-amyloid load. PHFtau was detected using an antibody specific for phosphorylated tau. Quantification of tangle density was accomplished using a stereological mapping station. Regional specific PHF tau tangle density (per mm^2^) was square root transformed and then averaged across brain regions to a summary measure for PHFtau tangle density.

**MS analysis of sera N-glycans**

5µl of serum samples were lyophilized, and N-glycans were separated using the enzyme rapidTM PNGAseF (New England Bioloabs, Ipswich, MA) following the manufacturer instruction and with one hour of enzyme incubation. The enzymatic reaction was stopped by the addition of 100µl of a 5% of acetic acid solution. N-glycans were purified and washed. Flow through and wash fraction were collected, pooled and lyophilized.

N-glycans permethylation was performed. Lyophilized N-glycan purified samples were incubated with 200µl of a DMSO (Dimethyl Sulfoxide; Sigma-Aldrich)-NaOH (Sigma-Aldrich) slurry solution and 100µl of methyl iodide (Sigma-Aldrich) for 20-30 min under vigorous shacking at room temperature. The reaction was stopped with 200µl of MilliQ water. Permethylated N-glycans were purified out by adding 200µl of Chloroform (Sigma-Aldrich). 800µl of Milli-Q water were then added and the mixture was vortexed to wash the chloroform fraction. The water was separated by centrifugation and discarded. The wash step was repeated 3 times. The chloroform fraction was dried and then redissolved in 200µl of 50% methanol prior to be loaded into a conditioned (1CV methanol, 1CV MiliQ water, 1 CV acetonitrile and 1 CV Milli-Q Water) C18 Sep-Pak (50 mg) column. The C18 column was washed with 3ml of 15% acetonitrile (Sigma-Aldrich) and then eluted with 3ml of 50% acetonitrile. The eluted fraction was lyophilized and then redissolved in 10µl of 75% methanol from which 1µl was mixed with 1µl DHB (2,5-dihydroxybenzoic acid) (5mg/ml in 50% acetonitrile with 0.1% trifluoroacetic acid (Sigma-Aldrich)) and spotted on a MALDI polished steel target plate (Bruker Daltonics, Bremen, Germany).

MS data was acquired on a Bruker UltraFlex II MALDI-TOF Mass Spectrometer using a reflective positive mode. Data between 500 m/z and 6000 m/z were recorded. For each MS N-glycan profiles, aggregates of 20,000 laser shots or more were considered for data extraction. Post-data acquisition analysis was conducted using mMass. MS signals that have a signal/noise ratio of at least 4 and match an N-glycan composition were included. The resulting sera glycan intensities was normalized using a global scaling. Briefly, individual sera glycan intensity was divided by the mean of sera glycan intensities within each sample, and then multiplied by the grand mean of glycan intensities across all samples. The normalized intensities were log2 transformed to reduce skewness and facilitate interpretation.

**MS analysis of cortical N-glycans**

20-100mg frozen dorsolateral prefrontal cortical tissue was prepared to quantify cortical N-glycans. After suspended in 1ml of lysis buffer (25 mM TRIS (Sigma-Aldricht, St Louis, MO), 150mM NaCl (Sigma-Aldricht), 5mM EDTA (Sigma, Aldricht), 0.5% w/v CHAPS (Sigma-Aldricht), pH 7.4), the lysed sample was dialyzed against 50 mM ammonium bicarbonate (Sigma-Aldricht) for 24h at 4ºC. The dialyzed material was lyophilized and resuspended in 1ml of a 2 mg/ml DTT (1,4-Dithiothreitol, Sigma-Aldricht) solution and incubated at 50ºC for 1h and 30min. 1ml of a 12mg/ml IAA (Iodoacetamide, Sigma-Aldricht) solution were then added and incubated at room temperature in the dark for 1h and 30min. The DTT and IAA treated sample was then dialyzed against 50mM ammonium bicarbonate as before. After lyophilization, the sample was resuspended in 1ml of 50µg/ml TPCK-treated trypsin (Sigma-Aldricht) solution and incubated at 37ºC overnight. The trypsin reaction was stopped with two drops of 5% acetic acid (Fisherbrand, Waltham, MA) prior to purification of the digested peptides over a C18 Sep-Pak (200mg) column (Waters, Milford, MA). Briefly, the Sep-Pak column was conditioned with 1 column volume (CV) of methanol (Sigma-Aldricht), 1 CV of 5% of acetic acid, 1 CV of 1-propanol (Sigma-Aldricht), and 1 CV of 5% of acetic acid. The trypsin-digested sample was then loaded onto the column and the column was washed with 6ml of 5% acetic acid. Peptides were eluted with 2ml of 20% 1-propanol in 5% acetic acid, then 2ml of 40% 1-propanol in 5% acetic acid and then 2ml of 100% 1-propanol. All fractions were pooled and subsequently lyophilized. The lyophilized peptides were resuspended in 200µl of 50mM ammonium bicarbonate to which 3µl of PNGaseF (New England Biolabs, Ipswich, MA) was added for a 4h-incubation at 37ºC. Following this initial incubation another 5µl of PNGaseF was added for overnight incubation at 37ºC. The enzymatic reaction was stopped by the addition of two drops of a 5% of acetic acid prior to the purification of the released N-glycans over a C18 Sep-Pak (200 mg) column conditioned as described above. The PNGaseF-treated sample was load onto the column and the column was washed with 1CV of 5% of acetic acid. Flow through and wash fraction containing the released N-gylcans were collected, pooled and lyophilized.

Lyophilized N-glycan samples were incubated with 1ml of a DMSO (Dimethyl Sulfoxide; Sigma)-NaOH (Sigma-Aldricht) slurry solution and 500µl of methyl iodide (Sigma-Aldricht) for 20-30 min under vigorous shacking. The reaction was stopped with 1ml of MilliQ water and 1ml of Chloroform (Sigma) was added to purify out the permethylated N-glycans. 3ml of Milli-Q water were added and the mixture was briefly vortexed to wash the chloroform fraction. The water was separated by centrifugation and discarded. This wash step was repeated 3 times and the chloroform fraction was finally dried before being redissolved in 200ml of 50% methanol prior to be loaded into a conditioned (1 CV methanol, 1 CV MilliQ water, 1 CV acetonitrile (Sigma) and 1 CV Milli-Q Water) C18 Sep-Pak (200 mg) column. The C18 column was washed with 6ml of 15% acetonitrile and then eluted with 6ml of 50% acetonitrile. The eluted fraction was lyophilized and then redissolved in 10µl of 75% methanol from which 1µl was mixed with 1µl DHB (2,5-dihydroxybenzoic acid) (50mg/ml in 50% acetonitrile with 0.1% trifluoroacetic acid (Sigma-Aldricht)) and spotted on a MALDI polished steel target plate (Bruker Daltonics, Bremen, Germany).

MS data was acquired on a Bruker UltraFlex II MALDI-TOF Mass Spectrometer using a reflective positive mode. Data between 1000 m/z and 6000 m/z were recorded. For each MS N-glycan profiles, aggregates of at least 20,000 laser shots were considered for data extraction. Subsequent MS post-data acquisition analysis was conducted using mMass. The MS profile is “smoothen” (using Savitzky-Golay method; 0.3 m/z window size; and 2 Cycles) and “baseline corrected” (using a Precision of 25 and a Relative offset of 25). Each MS profile is then recalibrated using major glycan peaks. Only MS signals matching N-glycan compositions were considered and reported for further analysis. Similar to sera glycans, cortical glycan intensities were globally scaled and log transformed for subsequent analysis.
