# Supplementary material for: Human Brain and Blood N-Glycome Profiling in Alzheimer’s Disease and Alzheimer’s Disease-Related Dementias
Source: Front Aging Neurosci. 2021 Oct 27;13:765259. doi: 10.3389/fnagi.2021.765259 (PMC8579010; doi:10.3389/fnagi.2021.765259)

**eFigure 1 Sera (A) and cortical (B) N-glycans by groups**


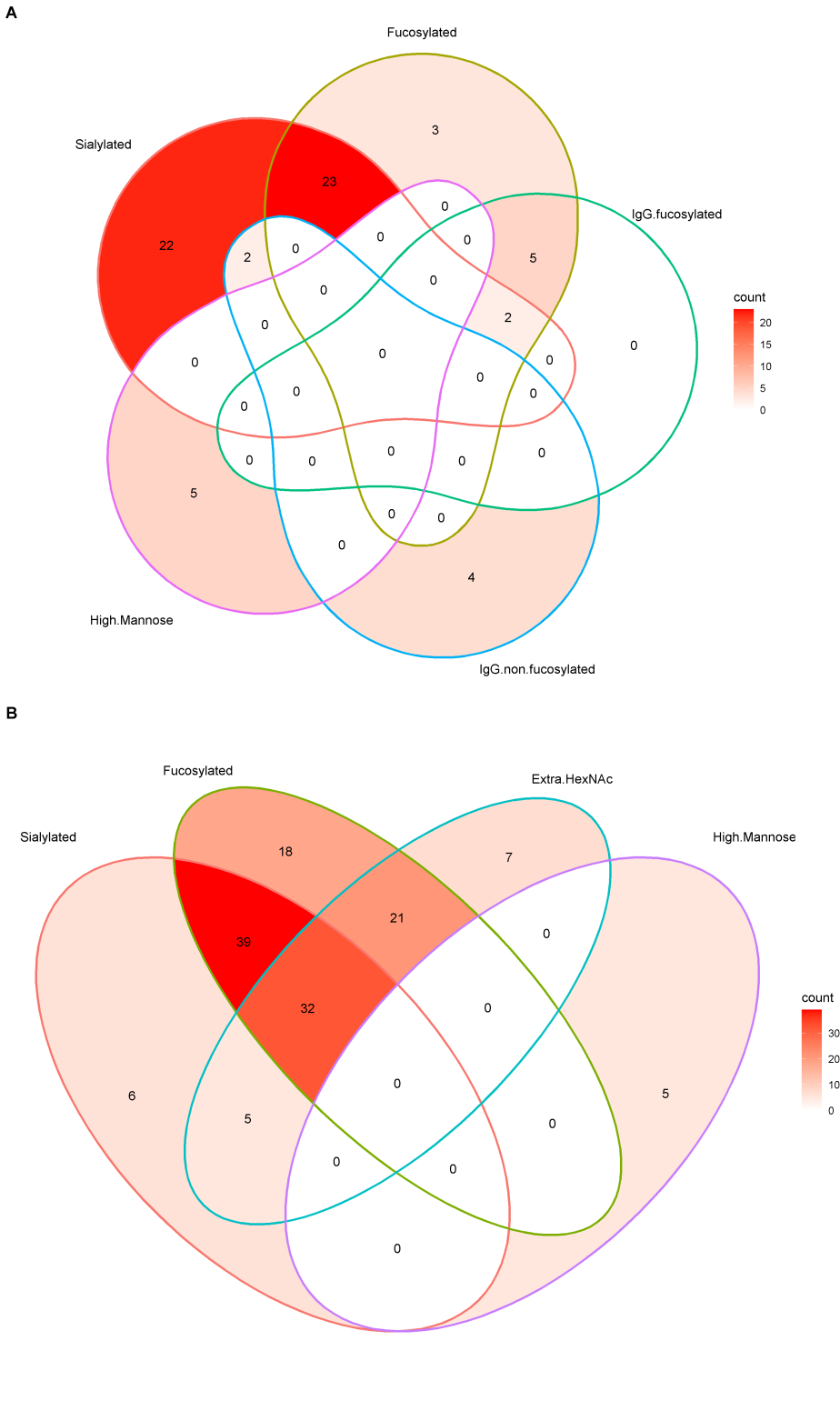


**eFigure 2 Clusters of sera N-glycans identified using am oblique principal component analysis**

Sera N-glycans at baseline


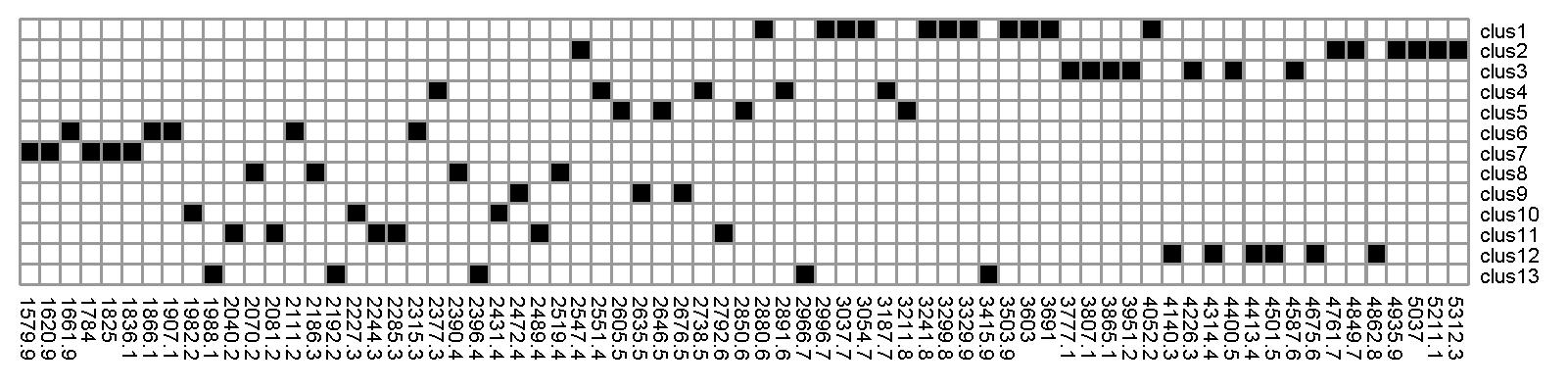


Sera N-glycans proximate to death


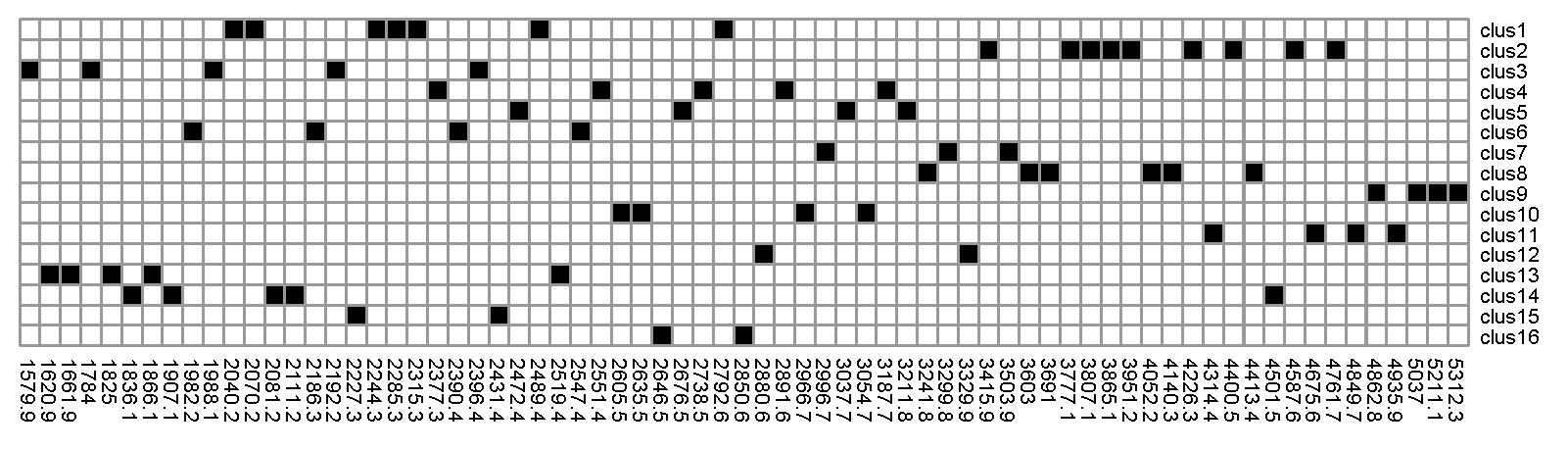


**eFigure 3 Paired correlations of sera N-Glycans between baseline and proximate to death**


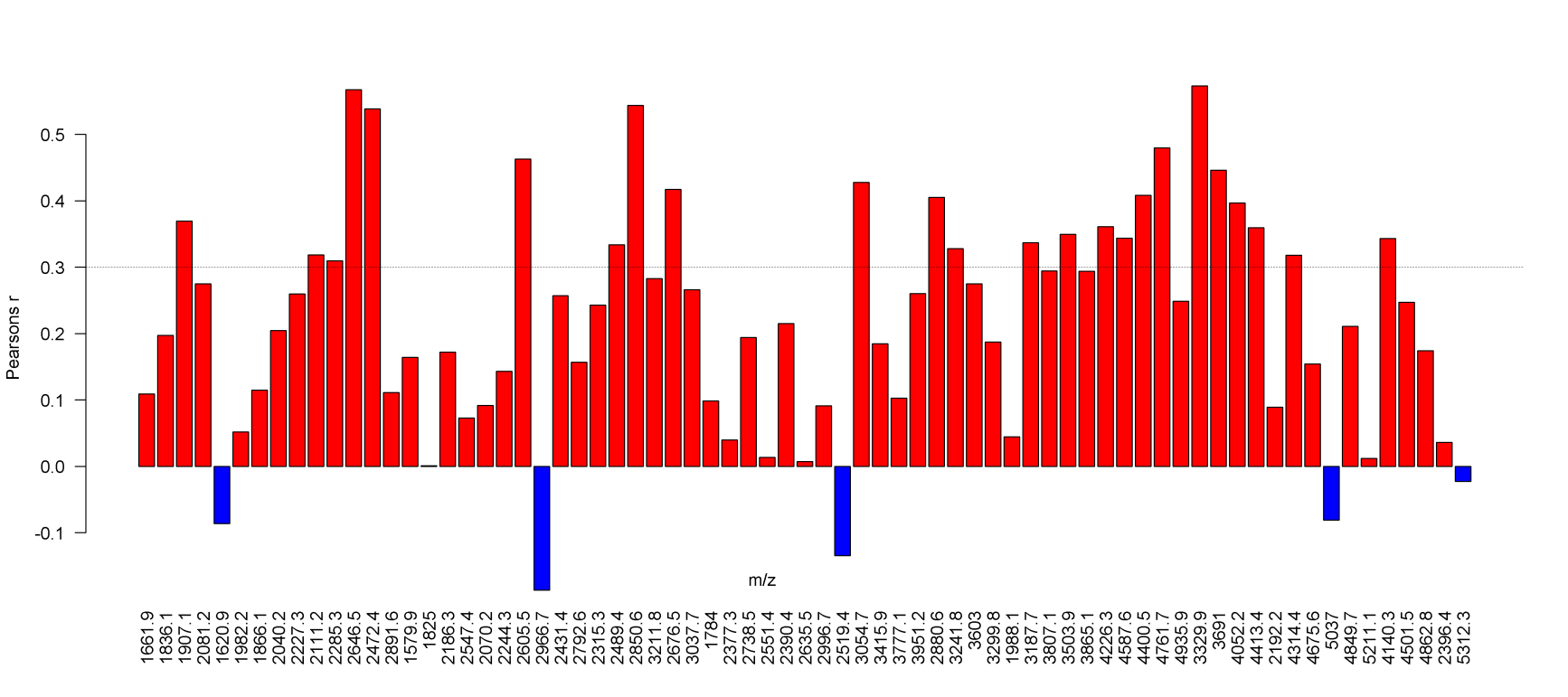


**eFigure 4 Correlations and clusters of cortical N-glycans**

**A B**


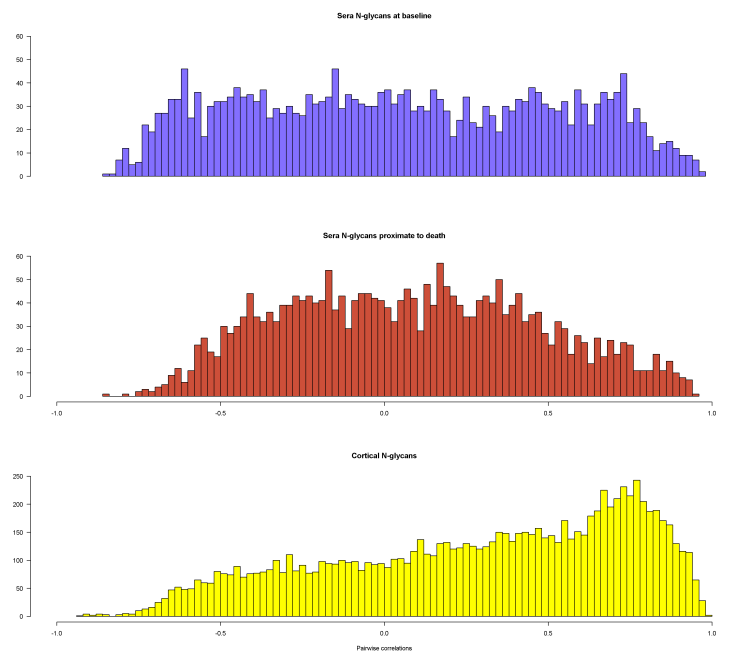

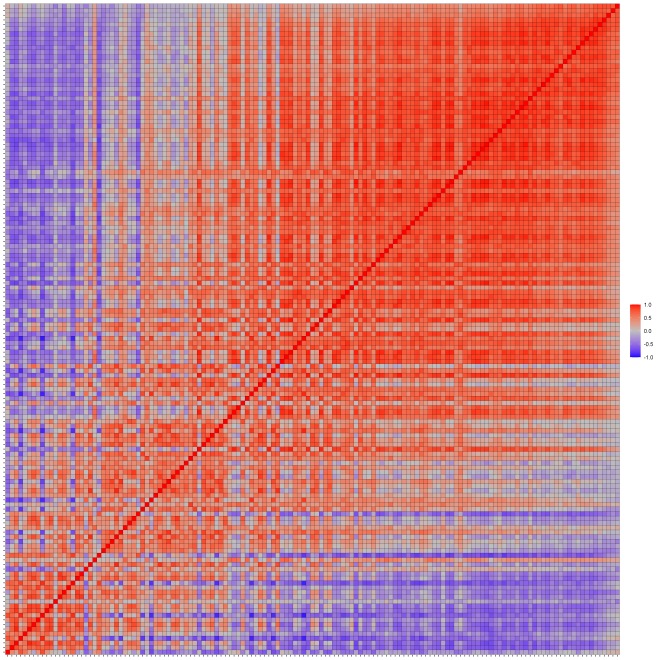


**C**


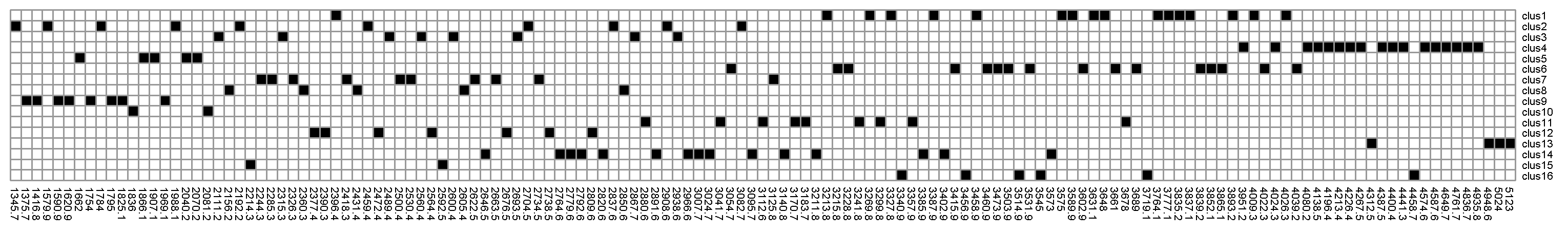


**eFigure 5 Correlations of N-glycans common in sera (proximate to death) and brain cortex**


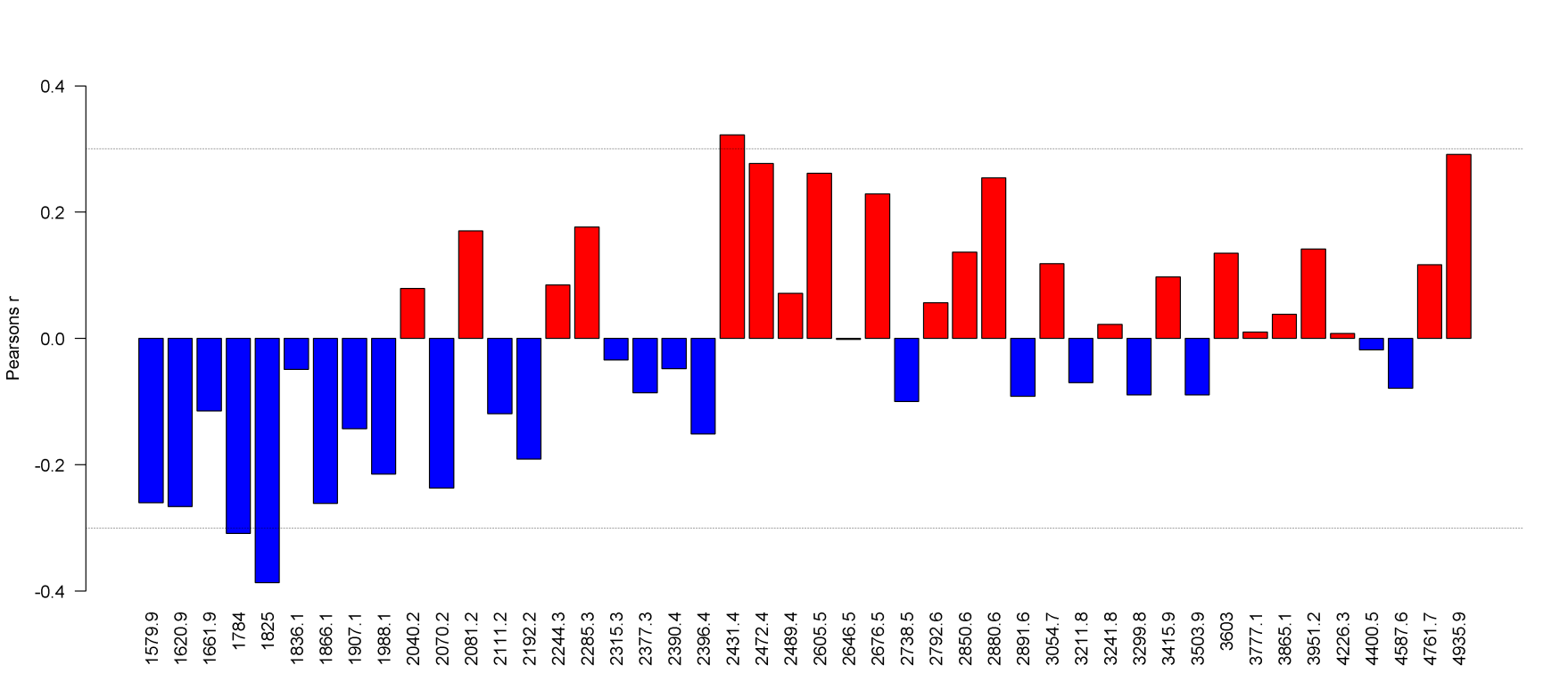


**eFigure 6 Expression levels of top baseline sera N-glycans associated with clinical diagnosis**

**
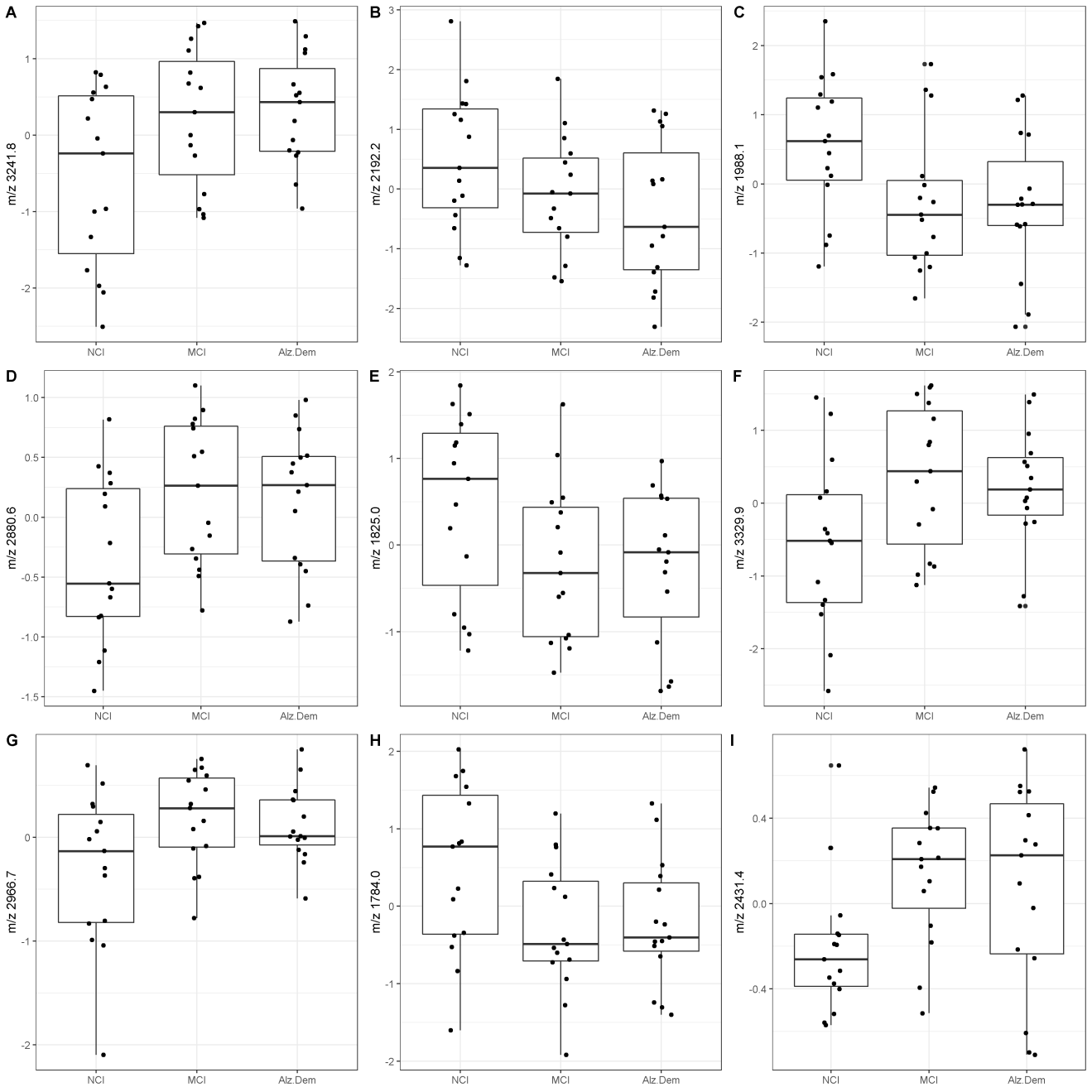
**

**eFigure 7 Cortical N-glycans and AD/ADRDs**


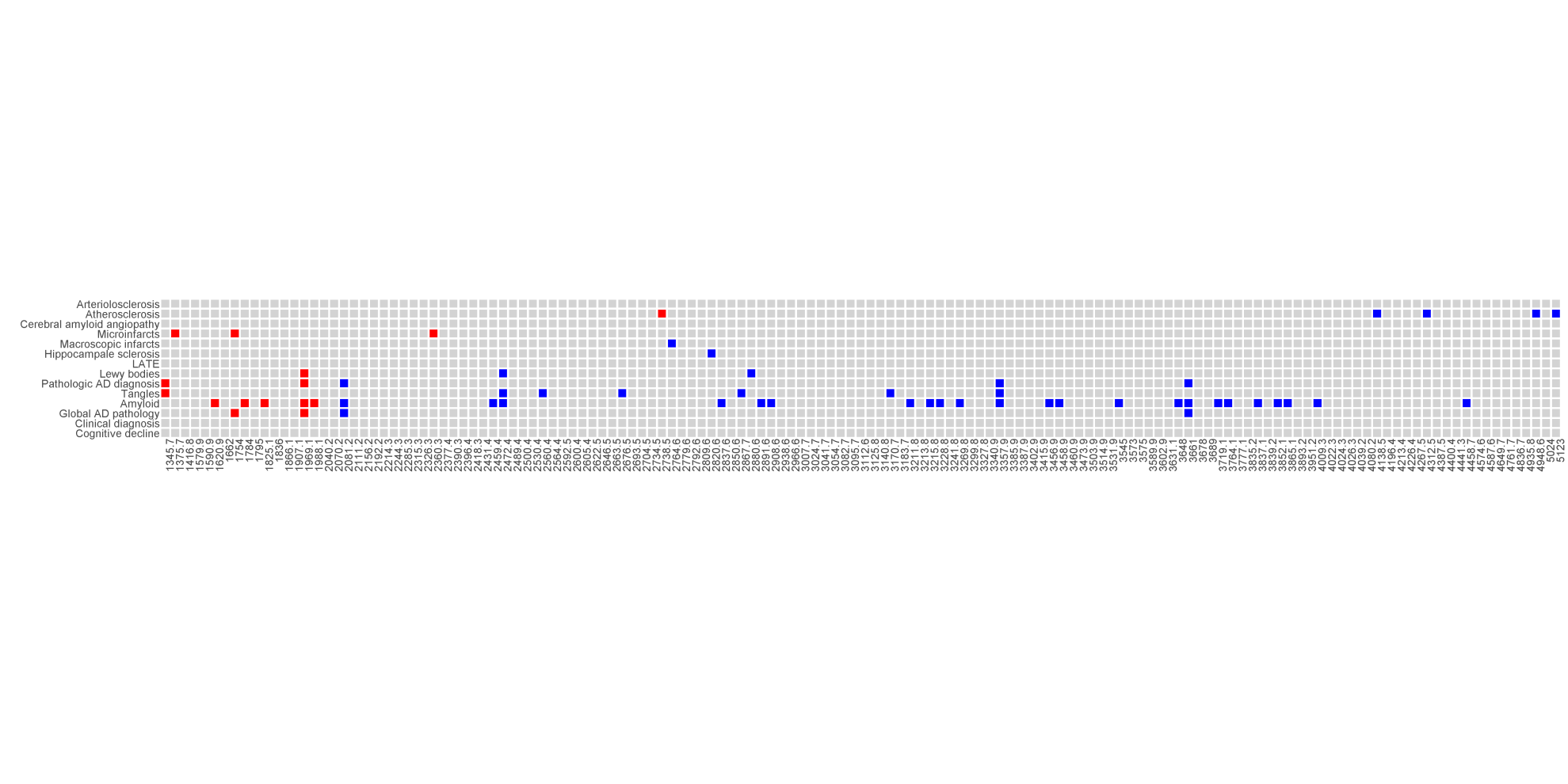

Supplement: Supplementary file 2 [file Data_Sheet_2.docx]
